# Zirconia-Reinforced Lithium Silicate Ceramic in Digital Dentistry: A Comprehensive Literature Review of Our Current Understanding

**DOI:** 10.3390/medicina59122135

**Published:** 2023-12-08

**Authors:** Manuela Manziuc, Andreea Kui, Andrea Chisnoiu, Anca Labuneț, Marius Negucioiu, Ana Ispas, Smaranda Buduru

**Affiliations:** Prosthetic Dentistry and Dental Materials Department, Iuliu Hatieganu University of Medicine and Pharmacy, 32 Clinicilor Street, 400006 Cluj-Napoca, Romania; manuelamanziuc@yahoo.com (M.M.); smarandabudurudana@gmail.com (S.B.)

**Keywords:** prosthetic dentistry, hybrid ceramic system, all-ceramic restorations, optical and mechanical properties, adhesion features

## Abstract

Zirconia-reinforced lithium silicate (ZLS) ceramic is a new innovative dental material with unique a chemical composition that is designed to combine harmoniously with the appropriate optical properties of lithium disilicate and the enhanced mechanical strength of zirconia. A thorough understanding of ZLS materials is essential for both clinicians and dental technicians. At present, the mechanical behavior and optical properties of the ZLS ceramic system have not been extensively researched, and there is still a lack of consensus regarding the fabrication process and clinical behavior of ZLS all-ceramic restorations. The aim of the present study was to present a selection of comprehensive information concerning zirconia-reinforced lithium silicate ceramics and their optical and mechanical properties, as well as to assess data regarding cementation procedures and clinical outcomes for ZLS all-ceramic restorations. Three electronic databases (PubMed, Web of Science, and the Cochrane Library) were used for the research by two independent reviewers. The search was limited to articles published in the English language, as well as clinical and in vitro studies of color and studies on mechanical behavior and the cementation procedures of ZLS restorations. The exclusion criteria comprised abstracts, questionnaire-based studies, case reports, literature reviews, and studies that were not available in English. Zirconia-reinforced lithium-silicate-based ceramic presents a unique and complex microstructure that increases mechanical resistance but decreases aesthetic appearance, especially its translucency, due to tetragonal zirconia content. A material’s thickness, the color of the underlying tooth structure, and the resin cement shade are important factors that influence the final shade and aesthetic appearance of ZLS restorations. Mechanical properties, which are defined by the fracture toughness, flexural strength, elastic modulus, and hardness of ZLS ceramic are higher compared to feldspathic, lithium disilicate, and hybrid ceramics, as well as resin nanoceramics; however, they are lower than translucent or high-translucency zirconia. Acid etching, sandblasting, and laser etching represent the most used methods to prepare the ZLS restoration surfaces for proper bonding procedures.

## 1. Introduction

In modern dentistry, the continuous development of computer-aided design (CAD) and computer-aided manufacturing (CAM) technology has gained significant popularity among dentists and dental technicians. This digital technology was introduced as an alternative to classic conventional manufacturing techniques to provide access to highly sophisticated tools for designing and fabricating a large diversity of dental restorations with a natural appearance, adequate mechanical resistance, and a higher level of precision [1].

At present, digital CAD/CAM systems are extremely efficient and widely used by dentists in their daily clinical practice. This new technology has resulted in the introduction of new types of CAD/CAM ceramic systems that have been exclusively created for this digital system [1,2].

To fulfill the increased aesthetic demands of their patients, clinicians prefer to use the new-generation, all-ceramic materials to create indirect restorations. Due to its increased capacity to mimic the optical properties of natural tooth structures, lithium-disilicate-based glass ceramic is one of the most desired restorative materials [2]. Unfortunately, the brittle structure of this type of ceramic system may limit its clinical indications and compromise the durability and performance of dental restorations [3].

In recent years, manufacturers have attempted to improve the strength of lithium-disilicate-based ceramic, introducing zirconia-reinforced lithium silicate (ZLS) ceramic, a new and innovative dental material with a unique chemical composition designed to harmoniously combine the appropriate optical properties of lithium disilicate with the enhanced mechanical strength of zirconia [4,5]. This new ceramic system consists of a lithium-metasilicate (Li_2_SiO_3_) glass–ceramic matrix that is reinforced with approximately 8–12% of zirconium dioxide grains (ZrO_2_), presenting a fine-grained microstructure (Li_2_O-ZrO_2_-SiO_2_) after the crystallization process [6,7]. Due to its increased translucency and color variety, ZLS can potentially be used to fabricate anatomical contours or monolithic restorations. Understanding the characteristics of ZLS ceramics is essential for clinicians and dental technicians to ensure the best treatment outcomes for their patients.

Currently, zirconia-reinforced lithium silicate (ZLS) ceramic is widely used for the fabrication of a large variety of all-ceramic restorations, being designed for CAD/CAM milling—Vita Suprinity PC (Vita Zahnfabrik, Bad Säckingen, Germany); Celtra Duo (Dentsply Sirona, Hanau-Wolfgang, Germany)—as well for pressing technique—Vita Ambria (Vita Zahnfabrik, Bad Säckingen, Germany); Celtra Press (Dentsply Sirona, Hanau-Wolfgang, Germany) [8,9,10].

Vita Suprinity PC (Vita Zahnfabrik, Bad Säckingen, Germany) is commercialized in a pre-crystallized form, allowing for easier milling of the restoration. However, due to the 10% weight content of zirconia, the material presents a unique homogeneous fine-particle architecture. Thus, after the crystallization process, the ceramic restorations present excellent optical and mechanical properties [8,10,11].

On the other hand, Celtra Duo (Dentsply Sirona, Hanau-Wolfgang, Germany) is manufactured in crystallized form and defined by a particular, ultra-fine microstructure due to the homogeneous distribution of zirconium dioxide grains in the glassy, amorphous matrix of lithium silicate crystals, which are four to eight times smaller than lithium disilicate crystals. The internal configuration of the glass–ceramic matrix, along with the very fine structure of the lithium silicate crystals, provides the material with particular mechanical and optical properties [8,12].

Zirconia-reinforced lithium silicate ceramics are indicated in a large variety of complex clinical situations, ranging from inlays, onlays, partial crowns, and veneers to anterior and posterior crowns and single-tooth restorations on implant abutments [13].

At present, clinical studies assessing the intraoral performance of ZLS restorations remain limited; however, their attributes align closely with those of lithium disilicate ceramics [14]. Consequently, in the context of medical education and practice, the primary aim of this literature review is to consolidate a comprehensive understanding of zirconia-reinforced lithium silicate ceramics, focusing on their optical and mechanical properties. In addition, we intended to evaluate existing data on cementation techniques and the clinical results associated with ZLS all-ceramic restorations, ensuring that emerging dental practitioners are familiar with contemporary advancements and methodologies. 

## 2. Materials and Methods

### 2.1. Search Strategy

This literature review was performed using the PRISMA standards [15]. The search was conducted between 1 July and 1 August using multiple databases, as follows: Medline (National Library of Medicine) via PubMed, Web of Science, and the Cochrane Library, including articles written in English with no timeframe. We optimized the search using Medical Subject Headings (MeSH) terms, in conjunction with specific keywords, all linked with Boolean operators (AND, OR). The exact terms and combinations of our search methodology can be found in Table 1. In addition, this review was registered in the PROSPERO database (registration ID: CRD42023469828).

### 2.2. Inclusion and Exclusion Criteria

The inclusion criteria were as follows: clinical and in vitro studies, articles published in English, studies assessing the optical and mechanical properties of ZLS milled restorations, studies assessing the cementation procedures and clinical outcome of this type of all-ceramic restoration. 

The exclusion criteria included: no full text available, survey studies or questionnaire-based studies, case reports, literature reviews, and articles published in languages other than English.

### 2.3. Study Selection and Data Extraction

In order to reduce bias, two independent reviewers (M.M. and A.K) selected data from each included article and recorded them in an Excel worksheet (version 15.17, Microsoft, Redmond, WA, USA). In case of disagreements/discrepancies, a third reviewer (B.S.) mediated a consensus. 

The following variables were defined in this investigation: first author’s last name, year of publication, study groups, sample size and thickness, study assessment, results.

### 2.4. Risk of Bias Assessment

The risk of bias assessment included the following domains: bias arising from the randomization process, bias due to deviations from intended interventions, bias due to missing outcome data, bias in the measurement of the outcome, bias in the selection of the reported result, and other bias. Based on the authors’ judgments, bias was classified as “low”, “high”, or “some concerns”. The quality of included studies was meticulously assessed using a systematic approach, focusing on clinical and in vitro studies that evaluated ZLS’s optical and mechanical properties, as well as cementation procedures and clinical outcomes. The review minimized selection bias through a detailed search strategy across multiple databases and employed standardized methods to mitigate detection bias. Both clinical and in vitro studies were included to reduce performance bias, ensuring a balanced perspective. The assessment process, involving two independent reviewers and a third for consensus in case of disagreements, had the purpose of reducing individual biases and attrition bias. To address reporting bias, a wide range of studies was included to ensure comprehensive coverage of ZLS research. As a result of this rigorous methodology, there was a significant reduction in the risk of bias, which enhanced the reliability and validity of the findings and allowed an unbiased understanding of ZLS in digital dentistry to be gained. This rigorous methodology significantly minimized the risk of bias, enhancing the reliability and validity of the findings and providing an unbiased understanding of ZLS in digital dentistry.

## 3. Results

**Figure 1:**
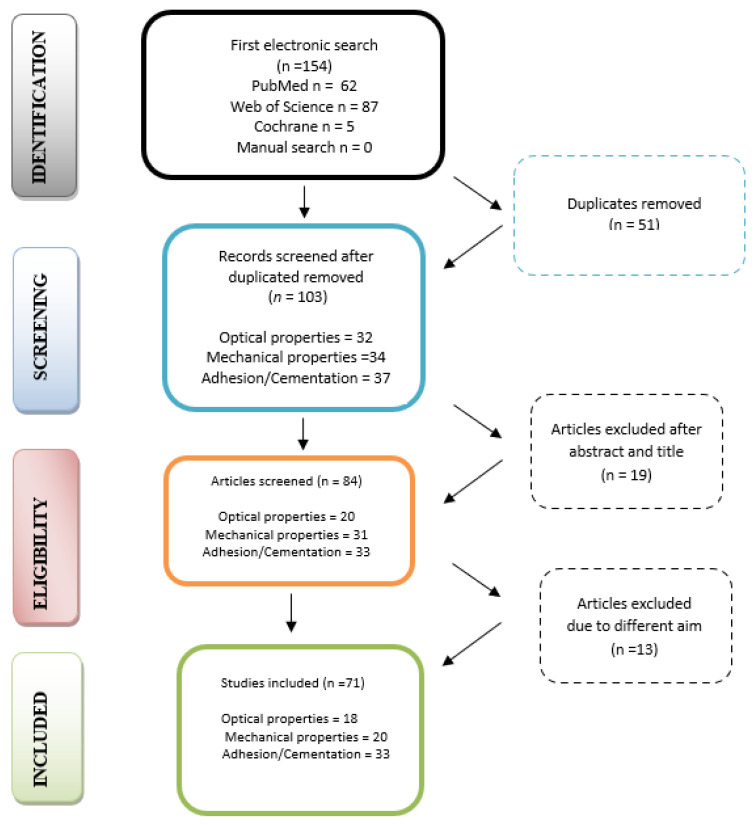
PRISMA flow diagram for research stages.

A total of 154 papers were found using the search method (PubMed = 62, Web of Science = 87, Cochrane = 5, manual search = 0). After removing the duplicates, 103 titles were reviewed and screened for eligibility. To identify the papers that were relevant to the aims of the research and in accordance with the inclusion criteria, the authors individually screened the abstracts. After the full text reading of the remaining studies, 32 publications were eliminated as they did not meet the inclusion criteria or did not match the outcomes of this paper (Figure 1). As a result, a total of 71 publications were included in this review (Tables S1–S3 in Supplementary Materials).

## 4. Discussion

### 4.1. Optical Properties of ZLS Ceramic Systems

When creating natural-looking restorations, choosing the accurate dental restorative material is one of the most important factors, as it should mimic to perfection the optical properties of the natural tooth structures [16]. A pleasant color integration of zirconia-reinforced lithium-silicate-based restorations is determined by the multifactorial interaction between the shades and thicknesses of the restorative material, the adhesive cement, the color of the underlying tooth structure, surface texture, and glaze [17,18,19,20].

In addition, the complex laboratory and manufacturing procedures will influence the final color of ZLS restorations, including their translucency [21,22,23]. Translucency is an essential characteristic of a ceramic material, defining its natural appearance [24]. However, chemical composition, the size and shape of the crystals, as well as their internal tridimensional configuration determine the clinical performance, shade, and translucency of ZLS restorations [25,26].

Zirconia-reinforced lithium silicate glass ceramic consists of quartz crystals (56–64%), lithium oxide (15–21%), alumina (1–4%), zirconium oxide (8–12%), potassium oxide (1–4%), and phosphorus pentoxide (3–8%) [27,28]. The scientific data indicate differences in microstructure between zirconia-reinforced lithium silicate (ZLS) and lithium disilicate (LDS) ceramics: (1) the size of lithium silicate crystals is approximately 0.5 μm compared to 1 μm, which is the size of lithium disilicate crystals [29]; (2) ZLS consists of two types of crystal structure, while LDS contains only one type of crystal. Thus, ZLS crystals possess a unique configuration, determining an increased mechanical resistance of the dental material, but with a decreased translucency, due to 8% to 12% zirconia content [30]. There is evidence in the literature showing 10wt% zirconia content has the most increased effect on the translucency parameter [31].

The effect of material and thickness on the translucency and color stability of zirconia-reinforced lithium silicate, lithium disilicate, and pre-shaded zirconia restorations, of 0.5, 0.7, and 1.0 mm thickness, was evaluated by Subasi et al. [32]. They concluded that the color of monolithic restorations was influenced by the material types, as well as by their thickness. For all the studied materials, the color changes were clinically acceptable, except for 0.5 mm ZLS. This indicates that at decreased thickness, the color of zirconia-reinforced lithium silicate restorations is likely to change. Nevertheless, the material’s thickness also affected the translucency; compared to pre-shaded zirconia, ZLS restorations showed increased translucency but were inferior to lithium disilicate restorations.

Furthermore, in a study published by Vichi et al. [33], the translucency of various factory-crystallized lithium silicate ceramics was compared to lithium disilicate ceramics and leucite glass ceramics, which require thermal treatment. Among the studied materials, zirconia-reinforced lithium silicate samples had the highest opacity, while lithium disilicate samples had the lowest.

The thickness of ceramic significantly influences the ultimate color and translucency of the ceramic restoration, while also playing a key role in concealing any discoloration of the underlying material. Passos and colleagues [34] conducted a study to assess the effectiveness of monolithic zirconia-reinforced lithium silicate (ZLS) restorations of varying thicknesses (1.0, 1.5, and 2.0 mm) and translucencies (high translucency (HT) and low translucency (LT)) in masking different substrates such as B1, C2, silver, and gold. The findings of their research indicated that a minimum ceramic thickness of 1.5 mm is necessary to effectively conceal the gold substrate, while a 2.0 mm thickness is essential for masking the C2 shade background in ceramic restorations. The study also found that none of the zirconia-reinforced lithium silicate (ZLS) restorations, irrespective of their thickness, were able to adequately mask the color of the silver background. Additionally, it was observed that restorations with a thickness of only 1.0 mm, regardless of the dental material used, did not yield satisfactory aesthetic results due to the color alterations caused by the underlying substrates.

Besides their ability to mask underlying colors, the color stability of ceramic restorations is crucial for their aesthetic integration and long-term success. Discoloration and loss of translucency, often caused by colorant beverages, can lead to significant patient dissatisfaction [35,36]. Aydin et al. [37] investigated the color changes in zirconia-reinforced lithium silicate (ZLS) CAD/CAM, composite, and hybrid ceramic restorations after exposure to various beverages. They found that red wine, followed by coffee, induced the most significant discoloration in all tested materials, exceeding clinically acceptable levels. However, ZLS CAD/CAM restorations exhibited the least color change, indicating superior color stability compared to resin-based dental materials. Other studies [38,39] have reported similar findings, but they also noted that ZLS ceramics have lower color stability compared to lithium disilicate. As the optical properties of dental materials could be influenced by smokeless tobacco, its effect upon the optical properties of ZLS restorations was investigated. According to Al Moallem et al. [40], the components of this product determined the highest color change in ZLS and feldspathic ceramic, and the least affected was multilayer zirconia ceramic.

Several studies have investigated the impact of aging on the optical characteristics of zirconia-reinforced lithium silicate (ZLS) ceramics. Alp et al. [41] proved that ZLS restorations with a thickness of 1.5 mm exhibit less translucency compared to those made from lithium disilicate, both before and after undergoing a coffee thermocycling aging process. This aging procedure significantly reduced the translucency of the restorations, irrespective of the type of dental material or the surface finishing technique used, whether glazed or polished. However, the study noted no discernible changes in color as a result of this process. In a study published by Arif et al. [38], the translucency of laminate veneers (0.7 mm) and crowns (1.3 to 1.5 mm) made from different ceramic systems was investigated. The authors concluded that coffee thermocycling impacts the translucency of zirconia-reinforced lithium silicate (ZLS) restorations. This finding contrasts with the results of Subasi et al. [32], who reported that coffee thermocycling does not influence the translucency of ZLS restorations. However, additional research has indicated that coffee thermocycling does indeed affect not only the translucency but also the opalescence of ZLS restorations [42,43,44].

Cakmak et al. [45] investigated the way different shades of resin cement (Tr, A2, and A3) and the thickness of the material (0.8 mm and 1.5 mm) influence the optical properties of zirconia-reinforced lithium silicate (ZLS) restorations, both before and after coffee thermocycling. Their findings revealed that both the shade of the resin cement and the thickness of the restoration material significantly impact the color of the restorations. The greatest translucency was observed in restorations of 0.8 mm thickness combined with Tr shade cement. The study also noted that the translucency of all tested restorations and cement shades diminished after exposure to coffee thermocycling. However, when exposed to ultraviolet irradiation, zirconia-reinforced lithium silicate glass ceramic exhibited the most significant decrease in translucency and increase in opacity [46]. In contrast, Comba et al. [47] investigated the effects of substrate and cement shade on the color properties of all-ceramic materials, concluding that the shade and translucency of lithium disilicate and zirconia restorations are heavily influenced by both intrinsic and extrinsic material properties, as well as the shade of the cement and the color of the substrate.

The translucency and optical properties of zirconia-reinforced lithium silicate (ZLS) all-ceramic restorations are primarily determined by the material’s intrinsic characteristics, such as its chemical composition, microstructure, and the size, shape, and distribution of crystals within the ceramic matrix [48,49]. However, laboratory and manufacturing processes, including firing temperature, vacuum duration, holding time, and the heating and cooling rates of the furnace [50], play a crucial role in defining the final aesthetic appearance of these restorations. Multiple firings can modify the crystalline structure of the restorations, leading to changes in color [49,51], and can also affect the value of optical parameters in ZLS restorations [52]. Nejatidanesh et al. [53] investigated the impact of multiple firings on the translucency of high-translucency (HT) and low-translucency (LT) lithium disilicate (LDS) and zirconia-reinforced lithium silicate glass ceramics, with thicknesses of 0.6 mm and 1.0 mm. The samples underwent three firing cycles (sintering, correction, and glaze firings) as per the manufacturer’s guidelines. Their findings indicated that these consecutive firing procedures did not affect the translucency of the 1.0 mm thick specimens but did cause alterations in the 0.6 mm thick specimens. Specifically, for the 0.6 mm thickness, the translucency of LT-LDS specimens increased, whereas the translucency of HT-ZLS specimens decreased. This phenomenon can be attributed to the zirconia content in ZLS ceramics, which negatively impacts the material’s translucency.

A study published by Schweitzer et al. [54] explored the impact of temperature variations on the optical properties of zirconia-reinforced lithium silicate (ZLS) ceramics. The ceramic samples were divided into three groups, each subjected to slightly different firing temperatures, minimally exceeding 820 °C. The findings revealed that increasing the temperature by 15 °C above the standard firing temperature recommended by the manufacturer significantly altered the optical properties of the ZLS specimens. This change manifested as an increase in brightness and a shift in the green–red coordinates towards red, and the blue–yellow coordinates towards yellow. Those findings are confirmed by a similar study published by Aurelio et al. [55] who observed a notable color change in ZLS ceramics. This suggests that ZLS ceramics are highly susceptible to color alterations, likely due to their lower glass content and higher proportion of fillers, such as metal oxides. These fillers can contribute to the color instability of this type of dental ceramic material.

The optical properties of zirconia-reinforced lithium silicate (ZLS) restorations are also influenced by surface finishing procedures, which determine their texture and roughness [56,57]. Common techniques for finishing the surfaces of restorations include glazing and mechanical polishing. Ozen et al. [58] found in their study that for ZLS ceramic restorations, manual polishing systems could serve as a viable alternative to glazing. This is due to the similar outcomes in optical parameters achieved by both finishing techniques. Furthermore, Kanat-Erturk et al. [39] observed that glazing enhances the color stability of ZLS ceramics. In contrast, Alp et al. [41] concluded that both glazing and polishing procedures do not significantly impact the color properties of ZLS restorations, with the differences being imperceptible. This suggests that the choice between glazing and polishing may be based more on preference or specific clinical considerations rather than significant differences in optical outcomes.

Despite the generally appealing aesthetic appearance of zirconia-reinforced lithium silicate (ZLS) glass ceramics, achieving perfect color integration of these ceramic restorations in certain clinical situations can be challenging. This difficulty arises from the various factors previously mentioned, each of which individually influences the translucency and color matching of ZLS restorations. These factors include the intrinsic properties of the material, such as its chemical composition and microstructure, as well as external influences like firing procedures, surface finishing techniques, and exposure to different environmental conditions. All these elements play a significant role in determining the final aesthetic outcome of ZLS restorations.

### 4.2. Mechanical Properties of ZLS Ceramic Systems

A zirconia-reinforced lithium silicate ceramic system is defined by a complex and unique microstructure, organized in a glassy matrix in which zirconia is embedded alongside lithium orthophosphate and lithium metasilicate crystals. This particular chemical composition in combination with the homogeneous distribution of tetragonal zirconia grains in the ceramic matrix leads to an increase in the mechanical properties of the ZLS ceramic system. Unlike lithium disilicate, ZLS exhibits an increased resistance to crack propagation, due to the capacity of zirconia grains to transform from a tetragonal to monoclinic phase [13]. The expansion of zirconia grains from 3% to 5% in volume creates compressive stress in the microstructure of the ceramic material, stopping the crack propagation [59].

Over the years, different studies investigated the mechanical behavior of ZLS restorations by analyzing the material’s physical properties, such as fracture toughness, flexural strength, elastic modulus, or hardness. The majority of in vitro studies have revealed that ZLS restorations exhibit better mechanical properties compared to lithium disilicate ceramics [13,59,60,61] but worse properties compared to translucent or high-translucency zirconia [62]. Abu-Izze et al. [63] conducted a study focusing on the fatigue strength of minimally invasive tabletop restorations. Their research revealed that hybrid ceramics with a thickness of 1.0 mm exhibited greater fatigue resistance compared to 0.5 mm thick zirconia-reinforced lithium silicate (ZLS) restorations. This enhanced durability in hybrid ceramics can be attributed to their polymeric microstructure, which, unlike ZLS, improves the material’s resistance to bending. Furthermore, hybrid ceramics possess an elastic modulus similar to that of dentin, enabling them to better withstand occlusal forces and resist crack propagation. In contrast, ZLS ceramic restorations tend to exhibit increased stress concentration at the ceramic–adhesive interface. This difference in material properties highlights the importance of considering the specific clinical application and the mechanical demands placed on restorations when choosing between hybrid ceramics and ZLS for dental procedures.

Mendoca et al. [64] investigated the mechanical behavior of the monolithic crowns fabricated from four different types of dental materials. The results indicate that lithium disilicate and ZLS restorations possess higher fracture strength compared to hybrid high-performance polymer composite resin and a hybrid polymer-infiltrated ceramic. Among these materials, ZLS restorations were identified as the hardest and stiffest. Elsaka et al. [59] found that zirconia-reinforced lithium silicate (ZLS) restorations outperform lithium disilicate in fracture toughness, flexural strength, elastic modulus, and hardness, with a lower brittleness index, indicating superior mechanical properties. This is attributed to zirconia’s transformation toughening mechanism, enhancing resistance to crack propagation. In contrast, Ramos et al. [13] reported similar fracture toughness between ZLS and lithium disilicate, concluding that the addition of zirconia did not stop the crack propagation into the ZLS microstructure.

Various studies have shown that the firing process affects the mechanical properties of zirconia-reinforced lithium silicate (ZLS). While it enhances the strength of the restorations [32], it also reduces the Weibull modulus, indicating a decrease in material homogeneity [52]. To address defects from the milling process, Aurelio et al. [55] suggested an extended glaze firing protocol involving a 15-min dwell and slow cooling in a closed furnace to 200 °C. This method resulted in compressive residual stress in the ZLS samples, reducing crack propagation, in contrast to the conventional protocol that led to tensile stresses. However, Campanelli de Morais et al. [52] found that lithium disilicate ceramics have better mechanical strength than ZLS, regardless of the number of firings (one to seven). Schweitzer et al. [54] also investigated the influence of minimal extended firing on ZLS’s mechanical properties, comparing polishing and glazing finishes. They noted that a second extended glaze firing with a minimal temperature increase of +15 °C improved ZLS’s flexural strength, but multiple extended firings reduced material homogeneity, potentially causing invisible pre-damage in the glass matrix.

When simulating the aging procedure, by coffee thermocycling, ZLS ceramic showed the highest biaxial flexural strength values, compared to lithium disilicate and advanced lithium disilicate glass ceramic. Yet, all tested ceramic systems showed biaxial flexural strength values higher than 300 MPa, being suitable to fabricate crowns or three-unit fixed partial dentures, that do not include the molars as abutments [42].

Particular caution is necessary when making intraoral adjustments to the occlusal morphology of monolithic zirconia-reinforced lithium silicate (ZLS) restorations; the adjustments may reduce their fracture strength, potentially compromising their structural integrity and durability. Dentists are advised to avoid these procedures due to the detrimental effects of carbide burs and the irregularities introduced by manual fissure deepening [34].

The thickness of all-ceramic restorations, including monolithic zirconia-reinforced lithium silicate (ZLS), plays a crucial role in their mechanical behavior and survival rate. Research has shown a direct correlation between the thickness of these restorations and their mechanical resistance; as the thickness decreases, the risk of failure in monolithic ZLS restorations increases [63,65]. 

In their study, Bergamo et al. [65] revealed that at the thickness of 1.0 mm and 1.5 mm, monolithic ZLS crowns had a greater resistance compared with 0.5 mm restorations. The restorations were tested under progressively increasing occlusal loads to mimic clinical mouth-motion fatigue. For crowns with thicknesses of 1.0 mm and 1.5 mm, there were no significant differences in fracture resistance at 200 N, 300 N, and 400 N, maintaining a survival probability of 90%. In contrast, restorations with a thickness of 0.5 mm exhibited markedly lower fracture resistance, with survival probabilities of 69%, 41%, and 19% at 200 N, 300 N, and 400 N, respectively. Regardless of thickness, all tested monolithic crowns ultimately failed due to bulk fractures. Other studies have similarly found that fabricating minimally invasive ZLS restorations with a thickness of 0.5 mm or less, particularly for molars, compromises their survival due to reduced mechanical resistance to occlusal loads [63,66].

The impact of acidic substances, like acid drinks or gastric acid, on the mechanical behavior of zirconia-reinforced lithium silicate (ZLS) ceramics was assessed in several studies. Picolo et al. [67] investigated how gastric acid erosion combined with mechanical toothbrushing abrasion affects the flexural strength, surface roughness, and microhardness of various dental materials, including ZLS, feldspathic glass ceramic, hybrid ceramic, and resin nanoceramic. The study found that feldspathic ceramic had the lowest flexural strength, primarily due to the acid solution’s effect on its silica glass content. In another study, ZLS and resin nanoceramic demonstrated the highest flexural strength, unaffected by either erosion or abrasion. This resilience in ZLS was attributed to its 10 wt% zirconium dioxide content, which limits crack propagation, while the 86 wt% inorganic concentration and 14% organic phase in resin nanoceramics contribute to their strength. The research revealed that exposure to erosive conditions, or a combination of erosive and abrasive conditions, reduced the microhardness of ZLS, feldspathic, and hybrid ceramics. In terms of biofilm adhesion, ZLS, feldspathic, and hybrid ceramics showed higher values compared to resin nanoceramic materials. Interestingly, when exposed to Coca-Cola, a popular carbonated beverage, ZLS ceramics exhibited the highest microhardness among the tested dental materials [68].

### 4.3. Cementation and Adhesion Features of ZLS Ceramic Systems

Failure of all-ceramic restorations may occur due to improper cementation processes. To achieve an accurate adhesion between the ceramics and the resin cement, chemical or micromechanical retentions are necessary in the inner surface of the restorations [68]. The conventional surface treatment of lithium disilicate restorations involves hydrofluoric acid etching that determines selective morphological changes of the glassy phase, by exposing silica, which chemically reacts with the silane-coupling agent to improve the bond between the resin cement and the ceramic material [69,70]. Due to enhancement with zirconia grains, ZLS ceramics may show a certain resistance to acid etching. Thus, several different surface treatment methods have been proposed and studied in order to establish the most adequate one, which enhances the shear bond strength of ZLS ceramics to resin cements [71]. Acid etching, sandblasting, and laser etching represent the most used methods to prepare the restorations surfaces for proper bonding procedures.

Acid etching is a commonly used procedure to enhance the adhesion of resin cement to dental ceramics, by creating microirregularities or micropores in the material’s inner surface [72].

Maawadh et al. [73] investigated the impact of four different etching durations on the bond strength of zirconia-reinforced lithium silicate (ZLS) restorations, using 9% hydrofluoric acid for etching times of 10, 20, 30, and 60 s. Their findings indicated that the optimal bond strength was achieved with an etching duration of 20–30 s. Extending the etching time beyond 30 s adversely affected the bond strength. Specifically, increasing the etching duration to 90 s led to a decrease in bond strength, attributed to reduced wettability of the ceramic surface [74]. Due to the presence of tetragonal zirconia, another study suggested that zirconia-reinforced lithium silicate (ZLS) ceramics might benefit from an additional surface treatment designed to increase the roughness of the restoration’s inner surface. Sandblasting has the capacity to increase the wettability and surface area, therefore strengthening the bond; however, while potentially beneficial for bonding, this may lead to the formation of microcracks which could contribute to the premature failure of the ceramic restorations [75]. Researchers have conducted thorough studies on how the size of alumina particles affects bond strength. They concluded that using particles larger than 50 μm leads to micromorphological changes in the ceramic material, negatively impacting adhesion. This is because larger particles create a weaker bond between the resin cement and the dental restoration, primarily due to the deterioration they cause in the dental ceramic [76]. Ataol et al. [74] evaluated the effect of the three surface treatment methods mentioned (etching with 9% hydrofluoric acid for 90 s, sandblasting by using 50 μm alumina particles for 20 s, and laser etching by using 2.94 μm wavelength, at 50 Hz for 140 μs), along with two bonding procedures (silane application and bond application). Their conclusions revealed that the highest bond strength was achieved for the restorations treated by etching. Their findings are consistent with other studies [71,77], which also reported that acid-etched samples exhibited the greatest increase in bond strength. This enhanced bonding can be attributed to the acid sensitivity of silica crystals in ZLS restorations, which improves micromechanical retention.

Azevedo et al. [78] treated ZLS and feldspathic glass ceramic restorations by using, consecutively, 5% and 10% hydrofluoric acid (HF) for 20, 40, and 60 s, while the control group was sandblasted with 50 μm aluminum oxide for 20 s. The obtained result indicated that the highest surface roughness was achieved by sandblasting the ZLS and 10% acid etching the feldspathic ceramic for 20 or 40 s. While increasing the roughness of the inner surface of a restoration can improve bonding, it is not always the optimal approach. Excessive roughness can lead to deep irregularities that may cause cracks in the ceramic microstructure, thereby reducing its mechanical resistance. Studies have indicated that increasing the concentration of hydrofluoric acid or prolonging the etching time does not necessarily improve bond strength; instead, it can diminish the mechanical resistance of glass ceramics [79]. Consequently, the recommended surface treatment for feldspathic ceramics is etching with 5% or 10% hydrofluoric acid for 20 s. For zirconia-reinforced lithium silicate (ZLS) ceramics, the preferred method is etching with 10% hydrofluoric acid for 40 s.

In their study, Altan et al. [71] concluded that treating zirconia-reinforced lithium silicate (ZLS) with laser irradiation (Er:YAG or Nd:YAG), either alone or in combination with sandblasting, enhances bond strength compared to acid etching alone. However, several studies have shown that the highest bond strength is achieved when the inner surfaces of the restorations are first treated with acid etching which is then followed by laser irradiation [71,77]. This sequential combination of acid etching and laser treatment is believed to significantly boost bond strength due to their synergistic effects.

Applying the silane onto the etched ceramic surface is an essential step in order to achieve a good bond strength, due to its unique chemical structure—the silanol component which interacts with the surface of the restorations and methacrylate component that copolymerizes with the resin cements [80]. Due to the incorporation of zirconia into the glassy matrix of zirconia-reinforced lithium silicate (ZLS) ceramics, the use of a bifunctional primer has been considered necessary. This primer is designed to effectively bond the two distinct structural phases present in ZLS ceramics: zirconia and silica. The bifunctional primer ensures adequate adhesion between these differing components, enhancing the overall integrity and durability of the restoration. Studies have shown that, unlike sandblasting, the acid-etching procedure generates an increased homogeneous roughness of the ZLS ceramic, which creates a higher bond strength [68,81]. Cinar et al. [68] evaluated the effect of the silane treatment on the bond strength of ZLS and lithium disilicate ceramics, as well as polymer-infiltrated ceramic, and concluded that acid etching (20 s and 60 s of 9.5% HF) and sandblasting (50 μm alumina particles, 2 bar pressure), followed by silanization, significantly improve the bond strength of all-ceramic restorations. For zirconia-reinforced lithium silicate (ZLS) ceramics, the optimal shear bond strength was obtained when the restorations were treated with hydrofluoric acid, followed by the application of silane. Additionally, it was observed that ZLS restorations exhibited higher bond strength compared to those made from lithium disilicate, regardless of the surface treatment method used, whether hydrofluoric acid alone or in combination with silane application. These findings align with other studies [82,83,84,85], which also concluded that the greatest shear bond strength for ZLS ceramics is achieved by treating them with hydrofluoric acid (either 5% or 9% for 20 s) followed by silane application.

In contrast, some studies have related that treating the ZLS ceramics by sandblasting with CoJet Sand^TM^ (3M, Maplewood, MN, USA), followed by silanization, results in superior microtensile bond strength for dental restorations. This method is found to be more effective compared to treatments involving etching with 5% hydrofluoric acid or etching followed by silane application. The enhanced bonding is attributed to the tribochemical silica-coating process, which effectively embeds silica particles into the ceramic matrix. This addition of silica particles strengthens the chemical bond among the three key components essential for optimal bond structure: the coated silica, silane, and composite resin [86]. Pucci et al. [87] introduced a new perspective by examining how the bonding process and aging affect the bond strength of polymer-infiltrated ceramic (PICN) and zirconia-reinforced lithium silicate (ZLS) ceramics. According to the manufacturer’s guidelines, PICN should be treated with 5% hydrofluoric acid (HF) for 60 s and then silanized for 60 s, while ZLS ceramics should be etched for 30 s and silanized for the same duration [9,88]. However, sandblasting with aluminum oxide not only creates a rough surface that enhances bond strength but also effectively removes residues from the restoration’s fabrication process. These residues, if not removed, could potentially interfere with the bonding procedure, making sandblasting a crucial step in preparing the surface for optimal adhesion. The authors tested ceramic samples with various treatments: 5% hydrofluoric acid etching plus silanization (HF + SI), air abrasion with 50 μm Al2O3 followed by HF etching and silanization (AB + HF + SI), HF etching plus universal adhesive (HF + UA), and air abrasion with Al2O3, HF etching, silanization, plus universal adhesive (AB + HF + SI + UA). Samples were stored in water for 24 h and one year, following manufacturer-recommended etching times for each material. Surprisingly, sandblasting did not enhance shear bond strength. Both PICN and ZLS showed no bond strength differences after 24 h in water but experienced decreased bond strength after one year. The study concluded that the highest bond strength after a year for PICN was achieved with HF + UA, and for ZLS with AB + HF + SI + UA.

Selecting the right luting cement is crucial for achieving optimal bond strength with zirconia-reinforced lithium silicate (ZLS) restorations. Self-adhesive or dual-cure resin cements are commonly chosen for cementing indirect restorations because of their user-friendliness and superior bonding capabilities [88]. Additionally, studies have identified MDP resin cement, which contains the adhesive monomer 10-methacryloyloxydecyl dihydrogen phosphate, as particularly effective for cementing ZLS restorations, owing to the presence of zirconia grains in their glassy matrix [68,83].

Okutan et al. [89] investigated the effect of the ceramic thickness and light polymerized resin cement on the shear bond strength of restorations; their conclusions showed that for all tested materials (leucite-based glass ceramic, a polymer-infiltrated ceramic, zirconia-reinforced lithium silicate glass ceramic), the 1 mm thick specimens showed the highest bond strength values, whereas 2 mm thick leucite glass ceramics and ZLS ceramics presented higher shear bond strength compared with 3 mm thick ones. Preis et al. [90] found that there was no significant difference in marginal adaptation between cemented ZLS crowns and their control counterparts. For successful clinical outcomes with ZLS restorations, the preparation design is of great importance, impacting their aesthetic, biological integration, and mechanical properties. Marginal and internal adaptations are crucial for the clinical performance and longevity of all-ceramic restorations. Falahchai et al. [91] examined the impact of finish line design on the marginal fit of ZLS restorations, testing four different preparation designs on natural teeth: anatomical occlusal reduction (O), with rounded shoulder (OS), with a central groove (OG), and with both rounded shoulder and central groove (OSG). They found that the marginal gap in all groups was <120 μm, both pre- and post-cementation, except for OSG, aligning with clinically accepted standards [92,93]. The best marginal adaptation was observed in the minimal invasive anatomical occlusal reduction design. As preparation complexity increased, achieving an ideal marginal fit became more challenging. The study also noted that the marginal gap widened by 36 to 37 μm post-cementation, highlighting the importance of careful cementation procedures. Other studies [94,95,96,97] concluded that cementation significantly impacts the marginal adaptation of ZLS restorations, with an increase in the vertical gap due to the thickness of the cement film. However, this vertical gap remained within clinically acceptable standards. Research into the mechanical behavior of monolithic restorations like crowns or endocrowns, bonded to dentin analogue materials or extracted teeth, showed that ZLS restorations exhibit higher fatigue resistance, fracture load, and retention compared to lithium disilicate but less than zirconia [97,98]. Prospective studies assessing the clinical performance of zirconia-reinforced lithium silicate restorations have reported promising results. One study noted a 94% survival rate over a 2-year follow-up, another observed a 98% success rate at 3 years, and a third study reported a 100% survival rate for pre-molar restorations and 69% for molar restorations at a 5-year follow-up [99,100,101]. These findings highlight the effectiveness and durability of ZLS restorations in clinical applications.

**Figure 2:**
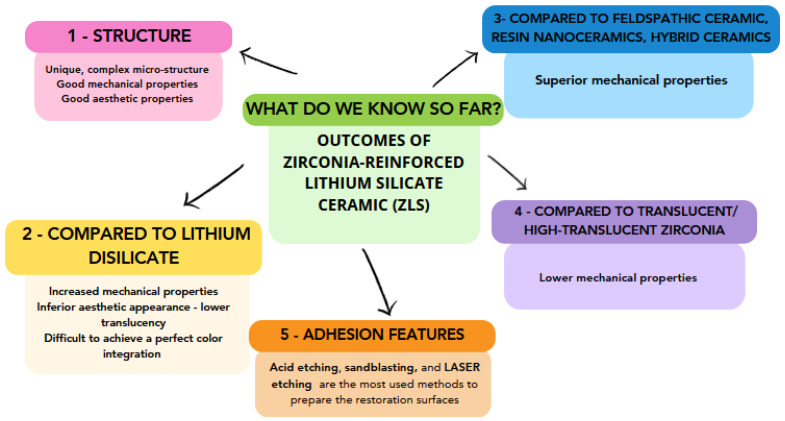
Overview of the outcomes of this integrative research regarding ZLS.

The continuous innovation and adaptation to evolving scenarios in dentistry are clearly reflected in the advancements of dental materials, particularly zirconia-reinforced lithium silicate (ZLS) ceramics (Figure 2). This progress is paralleled in the emerging field of teledentistry, which has gained prominence, especially in response to global challenges like the COVID-19 pandemic [102]. With the increasing focus on understanding the optimal application and characteristics of advanced materials such as ZLS, digital solutions for patient care are also on the rise. The rapid growth in teledentistry—its application across various dental disciplines—underscores this shift towards digitalization. However, much like the meticulous procedures required for ZLS restorations, teledentistry also has its limitations, emphasizing the need for a judicious blend of traditional and modern approaches. As our understanding of ZLS materials advances, it aligns with a broader narrative within the dental community, one that stresses ongoing education, adaptation, and a commitment to leveraging both material and technological advancements for optimal patient care.

## 5. Conclusions

Within the limitations of the present study, the literature review revealed the main characteristics and properties of zirconia-reinforced lithium-silicate-based ceramic, and the following conclusions could be drawn:

1. Unlike lithium disilicate glass ceramic, zirconia-reinforced lithium-silicate-based ceramic presents a unique, complex microstructure, which increases its mechanical resistance, but decreases its aesthetic appearance, especially its translucency, due to tetragonal zirconia content.
2. Over the years, the mechanical behavior of ZLS restorations has been widely studied and the results revealed that ZLS restorations exhibit better mechanical properties compared to feldspathic, lithium disilicate, and hybrid ceramics or resin nanoceramic but worse properties compared to translucent or high-translucency zirconia.
3. For accurate adhesion between the ZLS ceramics and the resin cement, chemical or micromechanical retentions must be created in the inner surface of the restorations.

## Figures and Tables

**Table 1:** Combination of search terms used in case of each database.

Search Terms and Combinations	
PubMed	“zirconia reinforced lithium silicate ceramic*”[tw] OR “zirconia reinforced lithium silicate glass-ceramic*”[tw] OR “ZLS”[tw] AND “Computer-Aided Design”[Mesh] OR “CAD-CAM*”[tw] AND “Optical Phenomena”[Mesh] OR “color*”[tw] OR “optical property*”[tw] OR “light scattering”[tw] OR “light transmission”[tw]
	“zirconia reinforced lithium silicate ceramic*”[tw] OR “zirconia reinforced lithium silicate glass-ceramic*”[tw] OR “ZLS”[tw] AND “Computer-Aided Design”[Mesh] OR “CAD-CAM*”[tw] AND “Mechanical Phenomena”[Mesh] AND “Flexural Strength”[Mesh] AND “Mechanical Tests”[Mesh] OR “mechanical performance”[tw] OR “flexural strength”[tw] OR “fatigue failure load”[tw] OR “surface hardness”[tw] OR “Young’s modulus”[tw]
	“zirconia reinforced lithium silicate ceramic*”[tw] OR “zirconia reinforced lithium silicate glass-ceramic*”[tw] OR “ZLS”[tw] AND “Computer-Aided Design”[Mesh] OR “CAD-CAM*”[tw] AND “Dental Bonding”[Mesh] AND “Acid Etching, Dental”[Mesh] OR “adhesion*”[tw] OR “surface treatment”[tw] OR “cementation*”[tw]
	“zirconia reinforced lithium silicate ceramic*”[tw] OR “zirconia reinforced lithium silicate glass-ceramic*”[tw] OR “ZLS”[tw] AND “Computer-Aided Design”[Mesh] OR “CAD-CAM*”[tw] AND “Clinical indication*”[tw] OR “Performances”[tw]
Web of Science	“zirconia reinforced lithium silicate ceramic” OR “ZLS” AND “Computer-Aided Design” OR “CAD-CAM” AND “Optical Phenomena” OR “color” OR “optical property” OR “light scattering” OR “light transmission”
	“zirconia reinforced lithium silicate ceramic” OR “ZLS” AND “Computer-Aided Design” OR “CAD-CAM” AND “Mechanical Phenomena” AND “Flexural Strength” AND “Mechanical Tests” OR “mechanical performance” OR “flexural strength” OR “fatigue failure load” OR “surface hardness” OR “Young’s modulus”
	“zirconia reinforced lithium silicate ceramic” OR “ZLS” AND “Computer-Aided Design” OR “CAD-CAM” AND “Dental Bonding” AND “Acid Etching, Dental” OR “adhesion” OR “surface treatment” OR “cementation”
	“zirconia reinforced lithium silicate ceramic” OR “zirconia reinforced lithium silicate glass-ceramic” OR “ZLS” AND “Computer-Aided Design” OR “CAD-CAM” AND “Clinical indication” OR “Performances”
Cochrane Library	“zirconia reinforced lithium silicate ceramic” OR “ZLS” AND “Computer-Aided Design” OR “CAD-CAM” AND OR “color” OR “optical property” OR “light transmission”
	“zirconia reinforced lithium silicate ceramic” OR “ZLS” AND “Computer-Aided Design” OR “CAD-CAM” AND “flexural strength” OR “fatigue failure load” OR “surface hardness” OR “Young’s modulus”
	“zirconia reinforced lithium silicate ceramic” OR “ZLS” AND “Computer-Aided Design” OR “CAD-CAM” AND “Acid Etching” OR “adhesion” OR “surface treatment” OR “cementation”
	“zirconia reinforced lithium silicate ceramic” OR “zirconia reinforced lithium silicate glass-ceramic” OR “ZLS” AND “Computer-Aided Design” OR “CAD-CAM” AND “Clinical indication” OR “Performances”

## Data Availability

All research data are in the supplementary files.

## Supplementary Materials

The following supporting information can be downloaded at: https://www.mdpi.com/article/10.3390/medicina59122135/s1, Table S1: Articles included based on mechanical parameters assessed; Table S2: Articles included based on aesthetic parameters assessed; Table S3: Articles included based on adhesion parameters assessed.
