# Supplementary material for: Zirconia-Reinforced Lithium Silicate Ceramic in Digital Dentistry: A Comprehensive Literature Review of Our Current Understanding
Source: Medicina (Kaunas). 2023 Dec 8;59(12):2135. doi: 10.3390/medicina59122135 (PMC10744576; doi:10.3390/medicina59122135)
Supplement: Supplementary file 1 [file medicina-59-02135-s001.zip › medicina-2742265-supplementary.pdf]

Table S1 – Articles included based on mechanical parameters assessed.

Study	Study Groups	Sample Size and Thickness	Parameters Assessed	Results
Ramos et al., 2016	• Vita Mark II (FEL)<br>• Vita Enamic (PIC)<br>• IPS e.max CAD (LD)<br>• Vita Suprinity (ZLS)	n = 15<br><br>• 12 mm diameter<br>• 1.2 mm thick	• Elastic modulus<br>• Poisson's ratio<br>• Fracture toughness<br>• Biaxial flexural strength (BFS)	• FEL and PIC, as well as LD and ZLS, showed similar fracture toughness<br>• The zirconia present in the ZLS did not improve the fracture toughness<br>• Weibull modulus of FEL, PIC, LD and ZLS was the same<br>• The FEL showed the lowest susceptibility to slow crack growth, as ZLS and LD had the highest
Elsaka et al., 2016	• Vita Suprinity (VS)<br>• IPS e.max CAD (IC)	n = 5<br><br>18 mm × 4 mm × 3 mm	• Fracture toughness Flexural strength<br>• Elastic modulus Hardness<br>• Brittleness index<br>• Microstructure	• VS showed higher fracture toughness, flexural strength, elastic modulus, and hardness than IC<br>• VS showed higher brittleness than IC<br>• VS had a homogeneous fine crystalline structure<br>• VS showed higher strength compared to IC

Study	Study Groups	Sample Size and Thickness	Parameters Assessed	Results
Vasiliu et al., 2022	• Vita PM9 (FP)<br>• Celtra Press (ZLSP)<br>• Vita Mark II (FM)<br>• Vita Suprinity (ZLSM)	n=8<br><br>Crown: 1.5 mm axial and occlusal reduction	• Fracture resistance and microhardness (VHN), before and after hydrothermal aging	• ZLD had higher microhardness, and fracture toughness compared to FP and FM<br>• Thermal aging effect the microhardness<br>• ZLSP and ZSLM showed no significant difference<br>• After thermal aging, ZLSM had the greatest microhardness, and FP lowest
Guilardi et al., 2020	• Vitablocs Mark II (FC)<br>• VITA Enamic (PICN)<br>• VITA Suprinity (ZLS)<br>• IPS e.max CAD (LD)<br>• VITA YZ (YZ)	n = 6<br><br>15 x 10 x 2 mm	• Impact strength	• FC showed the lowest impact strength, follow by PICN, LD, ZLS<br>• YZ had the highest impact strength
Attar et al., 2023	• IPS e.max CAD<br>• VITA Suprinity<br>• IPS Empress CAD<br>• Zenostar<br>• CopraSmile	n = 10<br><br>16 x 4 x 2 mm	• Flexural strength<br>• Weibull modulus	• Zenostar showed the highest flexural strength, while Empress CAD had the lowest<br>• Suprinity had the highest Weibull modulus, while Empress CAD showed the lowest

Study	Study Groups	Sample Size and Thickness	Parameters Assessed	Results
Abu-Izze et al., 2018	• Vita Suprinity (ZLS)<br>• Vita Enamic (PIC)	n= 15<br><br>0.5 and 1.0 mm	• Fatigue<br>• Failure mode<br>• Stress distribution	• 0.5 mm ZLS showed lower fatigue strength compared with 1.0 mm PIC<br>• PIC fractures were minor damage, less fragments compared with ZLS fractures<br>• ZLS showed higher concentrations of stresses in the adhesive interface
Mendoca et al., 2018	• IPS e.max CAD (LD)<br>• VITA Suprinity (ZLS)<br>• VITA Enamic (PICN)<br>• Cerasmart (HPP)	n = 10<br><br>Crown :<br>• 1.5 mm axial reduction<br>• 1.0 mm marginal chamfer<br>• 2.0 mm occlusal reduction	• Flexural strength<br>• Flexural modulus<br>• Fracture strength<br>• Microhardness<br>• Microstructure	• LD and ZLS had the highest fracture strength<br>• LD showed the highest flexural strength, then ZLS, HPP, PICN<br>• ZLS had the highest flexural modulus, then LD, PICN, HPP<br>• LD material showed small needle-shaped crystals<br>• ZLS had slightly larger crystals with more elongated, rounded, and rod-like appearance
Campanelli de Morais et al., 2020	• VITA Suprinity (ZLS)<br>• IPS e.max CAD (LD)	n=5<br><br>• 12 mm diameter	• Biaxial flexural strength (BFS)	• LD showed the highest BFS, regardless of the number of firings

Study	Study Groups	Sample Size and Thickness	Parameters Assessed	Results
		• 1.2 mm thick		
Aurelio et al., 2016	• VITABLOCS Mark II (FEL)<br>• IPS Empress CAD (LEU)<br>• IPS e.max CAD (DIS)<br>• VITA Suprinity (SLZ)	n=5<br><br>1.5 ± 0.5 mm	• The effect of extended glaze (EG) firing on crack healing and microstructure	• EG showed a more pronounced reduction in defect size, compared to conventional firing<br>• EG generated compressive stress into the material, increasing the fracture toughness<br>• After EG, ZLS crystalline peaks appeared more intense
Schweitzer et al., 2020	• Celtra Duo (ZLS)<br>• IPS e.max CAD (LDS)	n= 30<br><br>17 x 4 x 1 mm	• Flexural strength of the specimens subjected to polishing, standard firinging and three extended firings: the first (EF1) and second glaze firing (EF2) with controlled overheating of +15 C during the holding time	• Flexural strength showed non-normal distributions for EF1 and EF1+2<br>• Flexural strength for LDS and ZLS showed a significant difference<br>• EF2 increased ZLS flexural strength

Study	Study Groups	Sample Size and Thickness	Parameters Assessed	Results
Demirel et al., 2022	• CEREC Tessera (ALDS)<br>• IPS e.max CAD (LDS)<br>• VITA Suprinity (ZLS)	n = 10<br><br>• 12 mm diameter<br>• 1.2 mm thick	• Biaxial flexural strength (BFS), before and after 5000 cycles of coffee thermocycling	• ZLS showed the highest BFS<br>• LDS had similar BFS values to those of ALDS
Passos et al., 2018	• Vita Mark II<br>• IPS Empress CAD<br>• IPS e.max CAD<br>• Vita Suprinity PC	n = 10<br><br>Molar crown:<br>• 1.5 mm axial and occlusal<br>• 1.0 mm shoulder	• Fracture strength of:<br>• MEOM crowns - Finished without manual enhancement of occlusal morphology<br>• With MEOM crowns	• After MEOM crowns showed a decrease strength to fracture load - 28.4% (Vita Mark II), 41.1% (IPS Empress CAD), 45% (IPS e.max CAD), and 17.7% (Suprinity PC)<br>• Vita Suprinity showed mainly fracture through the crowns
Bergamo et al., 2019	• Vita Suprinity (ZLS)	n = 21<br><br>0.5, 1.0 and 1. 5mm	• Reliability and failure mode	• 0.5 mm ZLS had reduction in the reliability of 69% at 200N, 41% at 300 N, 19% at 400 N<br>• 1.0 and 1.5 mm ZLS had reduction in the reliability of 90% at 200N<br>• at 300 and 400N, 1.0 mm (95% and 86%) and 1.5mm crowns (99% and 94%)<br>• The main failure mode was the comprised bulk fracture

Study	Study Groups	Sample Size and Thickness	Parameters Assessed	Results
Monteiro et al, 2018	• Vita Suprinity (VS)<br>• Celtra Duo (CD)	n = 25<br><br>1.0, 1.5, 2.0 and 2.5 mm	• Fatigue failure load	• VS and CD showed an increase in mean fatigue failure load with an increase in ceramic thickness<br>• 2.5 mm VS and CD showed the highest fatigue failure load
Picolo et al., 2022	• Celtra Duo (ZLS)<br>• Vita Blocks Mark II (FE)<br>• Vita Enamic (PICN)<br>• Katana Avencia Block (RK)<br>• Grandio Blocs (RG)	n = 10<br><br>14 × 14 × 1 mm	• Flexural strength and microhardness of specimens exposed to erosion and/or abrasion	• FE showed the lowest flexure strength after erosion (E) and erosion and abrasion (E + A) simulations<br>• The acid-erosive exposure decreased microhardness of ZLS, FE, and PICN<br>• ZLS, FE, and PICN showed higher bio-film adhesion than RG and RK
Colombo et al., 2019	• Cerasmart<br>• Lava Ultimate<br>• Gradio Blocs<br>• VITA Suprinity PC	n = 15<br><br>2.0 mm	• Microhardness of specimens, before and after exposure to a carbonated acidic drink, Coca-Cola	• Both, in the control group and after 28 days of exposure to Cola , ZLS showed the highest microhardness<br>• In the control group, hybrid ceramic showed the lowest microhardness<br>• After 21 days of exposure, nano hybrid composite

Study	Study Groups	Sample Size and Thickness	Parameters Assessed	Results
				had the highest microhardness loss

Table S2 - Articles included based on esthetic parameters assessed

**Study**	**Study Groups**	**Sample Size and Thickness**	**Study Assessment**	**Results**
Subasi et al., 2018	• VITA Suprinity PC (ZLS)<br>• IPS e.max CAD (LDS)<br>• InCoris TZI C (MonZr)	n=4<br><br>0.5, 0.7, 1 mm	• Color difference (ΔE_{00}) and relative translucency parameter (RTP) were assessed by using a spectroradiometer, before and after, 5,000 coffee thermocycling cycles	• Coffee thermocycling did nor effect the translucency<br>• Thickness effected the ZLS color change<br>• MonZr had the smallest cooler change, not perceptible at any thickness
Passos et al.,	• Celtra Duo (ZLS) both,<br>HT - High - Translucency<br>LT - Low - Translucency	n = 15<br><br>1.0, 1.5 and 2.0 mm	• Color coordinates of each specimen were measured with a spectrophotometer<br>• The ΔE was calculated to determine the color difference between B1 and C2, silver and gold substrate	• C2 and silver can be make with 2.0 mm thickness<br>• Gold showed clinically acceptable values for 1.5 mm thickness
Sarikaya et al., 2018	• IPS e.max CAD (LDC)<br>• VITA Suprinity (ZLS)	n = 10<br><br>14 mm diameter 1.2 mm thick	• Color difference was assessed by using the spectrophotometer, before and after, immersing the specimens in tea, coffee and cola	• ΔE of ZLS were higher than LDC<br>• Coffee was the most colorant beverage

Study	Study Groups	Sample Size and Thicknes s	Study Assessment	Results
Aydin et al., 2019	• Cerasmart (hybrid ceramic)<br>• Shofu Block (hybrid ceramic)<br>• Grandio Blocs (composite)<br>• Brilliant Crios (composite)<br>• Celtra Duo (ZLS)	n=40<br><br>1.5 X 7 X 12 mm	• Color difference was measured by using spectrophotometer<br>• The used beverages were wine, coffee, cola, energy drink, distilled water<br>• Samples were stored in an incubator for 30 days, at 37oC	• Red wine and coffee caused the greatest color change on all tested materials (ΔE_{00}: 2.25), especially effecting the composite-containing materials<br>• Cola and energy drink effected the least the color (ΔE_{00}:1.30)
Arif et al., 2018	• Celtra Duo (CD)<br>• IPS e.max CAD (EM)<br>• Lava Ultimate (LU)<br>• VITA Enamic (VE)<br>• VITA Suprinity (VS)<br>• VITA YZ (Zir)	n=5<br><br>Laminate: 0.7 mm<br><br>Crown: 1.3, 1.5 mm	• Color difference and translucency (RTP) were assessed by using spectroradiometer, before and after 6,000 coffee thermocycling cycles	• LU presented the greatest color change<br>• EM was the most color stable<br>• Crowns showed lower translucency
Kanat-Erturk et al., 2019	• IPS e.max CAD HT<br>• Vita Suprinity HT	n=10<br><br>1.5 × 7 × 12 mm	• Color values were measured with spectrophotometer<br>• Color difference of glaze, mechanical polishing, and external staining and glaze specimens was assessed before and after coffee and black tea storage	• Lithium disilicate showed higher color stability, except for the external staining and glaze specimens storage in coffee<br>• Glazed specimens showed the highest color stability<br>• Polishing paste decreased the color variations

Study	Study Groups	Sample Size and Thickness	Study Assessment	Results
Moaleem et al., 2022	• Ceramill Zolid PS<br>• Vitablocs Vita Triluxe Forte<br>• VITA Suprinity	n=10<br><br>2, 12 and 14 mm	• Color change was assessed by using the spectrophotometer<br>• The specimens were immersed and stained in smokeless tobacco	• Zirconia showed the lowest color change, after one week<br>• Vita Suprinity and Ceramill Zolid showed the highest color change after tobacco exposure at one and two weeks
Alp et al., 2018	• VITA Suprinity PC (ZLS)<br>• IPS e.max CAD (LDS)	n=18<br><br>• rectangular plates<br>• 1.5 mm thick	• Color differences and relative translucency were assessed by using a noncontact spectroradiometer<br>• The glazed and polished samples were subjected to 5,000 thermocycles in a coffee solution	• Coffe thermocycling effected the color of ZLS and LDS<br>• LDS is more translucent than ZLS, before and after aging<br>• Coffee thermocycling decreased the translucency of both materials
Demirel et al., 2022	• CEREC Tessera (ALDS)<br>• IPS e.max CAD (LDS)<br>• VITA Suprinity (ZLS)	n = 10<br><br>• 12 mm diameter<br>• 1.2 mm thick	• Color differences (ΔE00) and relative translucency parameter (RTP) were assessed by using the digital spectrophotometer, before and after 5,000 cycles of coffee thermocycling	• All materials had similar color changes<br>• LDS had the highest and ZLS had the lowest RTP values, both before and after thermocycling<br>• Only LDS showed the same translucency before and after thermocycling

Study	Study Groups	Sample Size and Thicknes s	Study Assessment	Results
Vasiliu et al., 2020	• Vita PM9 (FP)<br>• IPS e.max Press (LDS)<br>• Celtra Press (ZLS)<br>• Vita Mark II (F)<br>• IPS Emax CAD (LD)<br>• Vita Suprinity (ZLS)	n=8<br><br>• rectang ular plates<br>• 1.5 mm thick	• Translucency (TP) and opalescence (OP) of polished and glazed samples were assessed by using a spectrophotometer, before and after 10,000 thermocycles in distilled water	• ZLS and FP were the most effected by the ageing process<br>• Milled and pressed ZLS and LDS showed significant change in opalescence and translucency after ageing<br>• Milled ZLS showed less translucency loss compared to pressed ZLS<br>• Thermocycling process effected the pressed glazed samples, but also the milled polished samples
Donmez et al., 2022	• Amber Mill (NLD)<br>• IPS e.max CAD (LDS)<br>• Vita Suprinity (ZLS)<br>• BRILLIANT Crios	n = 5<br><br>0.7 and 1.5 mm	• Color differences (ΔE00) and relative translucency parameter (RTP) were assessed by using a spectrophotometer, before and after 5,000 thermocycles in coffee	• 0.7 mm ZLS had the lowest ΔE00<br>• 1.5 mm LDS had higher ΔE than ZLS<br>• NLD had similar ΔE00 values to LDS and ZLS<br>• After thermocycling, RTP of 0.7 mm ZLS were lower than NLD and LDS<br>• LDS and NLD showed similar RTP, regardless of the thickness and aging process

Study	Study Groups	Sample Size and Thickness	Study Assessment	Results
Cakmak et al., 2021	• VITA Suprinity (ZLS)	n=5<br><br>0.8 and 1.5 mm	• Color difference (ΔE00) and relative translucency parameter (RTP) were assessed by using a noncontact spectroradiometer, before and after, 5,000 coffee thermocycling cycles (CTC)	• Translucency was effected by thickness, resin cement shade and coffee thermocycling<br>• Translucency decreased after CTC<br>• Color difference was effected by material thickness
Choi et al., 2020	• Tetric CAD (TC)<br>• Vitablocs Mark II (VM)<br>• IPS Empress CAD (EP)<br>• IPS e.max CAD (EM)<br>• Celtra Duo (CD)<br>• Vita Enamic (VE)<br>• Cerasmart (CS)<br>• Lava Ultimate (LU)	n = 15<br><br>14×12×1 mm<br><br>14×12×2 mm	• Color difference (ΔE00), translucency parameter (TP), total transmittance (Tt) and contrast ratio (CR) were assessed by using a spectrophotometer, before and after UV aging	• CD showed the lowest translucency and highest opacity<br>• TC showed the highest translucency<br>• CD showed the highest CR value and TC the lowest<br>• Ageing, material type and thickness influenced the optical properties of all tested materials

Study	Study Groups	Sample Size and Thicknes s	Study Assessment	Results
Campa nelli de Morais et al., 2020	• VITA Suprinity (ZLS)<br>• IPS e.max CAD (LD)	n=5<br><br>• 12 mm diamete r<br>• 1.2 mm thick	• Color and translucency variations were determined by using spectrophotometry<br>• The specimens were subjected to two firings (2F), five firings (5F) and seven firings (7F)	• For ZLS, 2F and 5 F effected the L* and b* values; L* was higher at 5F and 7F<br>• ZLS had higher L* than LD<br>• LD had higher a* than ZLS, for all the firing types<br>• ΔE of LD was significantly lower than ZLS<br>• TP had different values for LD and ZLS, at 2F and 5F, but simmer values at 7F<br>• ZLS showed higher TP at 7F
Nejatid anesh et al., 2019	• IPS e.max CAD (LDS)<br>• VITA Suprinity PC (ZLS)<br>both,<br>HT - High - Translucency<br>LT - Low - Translucency	n=10<br><br>0.6, 1 mm	• Translucency and contrast ratio were assessed by using a spectrophotometer<br>• The samples were subjected to crystallization, correction, and glaze firing cycles	• At 0.6 mm thickness, the translucency of both, HT-ZLS and LT-LDS, was effected by repeated firings<br>• Translusency increased as the thickness of the samples decreased

Study	Study Groups	Sample Size and Thicknes s	Study Assessment	Results
Schweitzer et al., 2020	• Celtra Duo (ZLS)<br>• IPS e.max CAD (LDS)	n= 30<br><br>17 x 4 x 1 mm	• Color was measured with a digital spectrophotometer, before and after firing<br>• specimens were subjected to polishing, standard firinging and three extended firings: the first (EF1) and second glaze firing (EF2) with controlled overheating of +15 C during the holding time	• The color change after firing of ZLS exceeded the perceptible threshold of ΔE >_1.5 in 23% of EF1 and 10% of EF2 specimens<br>• Between the polished experiments groups, L*a*b* values showed no difference
Aurelio et al., 2016	• VITABLOCS Mark II (FEL)<br>• IPS Empress CAD (LEU)<br>• IPS e.max CAD (DIS)<br>• VITA Suprinity (SLZ)	n=5<br><br>1.5 ± 0.5 mm	• Color difference and contrast ratio (CR) were assessed by using a spectrophotometer;<br>• Samples were subjected to conventional (G) firings and extended (EG) glaze firings: dwell time of 15 min, slow cooling rate	G and EG firings changed significantly:<br>• FEL: L* (EG) and a* (G)<br>• LEU: L*, a* (EG) and a* (G)<br>• DIS: L*, a*, b*, CR<br>• ZLS: L*, a*, CR
Ozen et al., 2020	• IPS e.max CAD (EC)<br>• VITA Suprinity (VS)<br>• LAVA Ultimate (LU)	n=24<br><br>14 x 12 x 0.5 mm	• Color change was assessed by using a spectrophotometer<br>• Samples were subjected to 5,000 cycles of artificial ageing before the treatment, after polishing, after cement application, and after artificial ageing	• LU exhibited higher color change than VS and EC, after artificial ageing.<br>• Polishing and glazing determined color change for LU and VS<br>• Color change of glazed VS and EC was below the clinically acceptable level (ΔE < 3.5)

Table S3 - Articles included based on adhesion parameters assessed

Study	Study Groups	Surface treatments	Sample Size and Thicknes s	Parameters Assessed	Results
Cinar et al., 2019	• IPS e.max CAD<br>• Vita Suprinity<br>• Lava Ultimate<br>• Vita Enamic	• Control<br>• Etching (9.5% HF, 20s glass /60s resin)<br>• Etching + silanization (HF + S)<br>• Sandblasting (SB 50μm aluminum oxide, 2 bar, 10 s, 10 mm)<br>• Sandblasting + silanization (SB + S)<br>• Self-adhesive resin cement	n=10<br><br>2.5 mm slices	• Shear bond strength (SBS)	• Vita Enamic showed the highest SBS<br>• Lava Ultimate had the highest SBS when using SB + S<br>• IPS e.max CAD, Vita Suprinity, and Vita Enamic showed the highest SBS, by using HF + S
Altan et al., 2019	• Vita YZ HT<br>• inCoris TZI<br>• IPS e.max ZirCAD<br>• Vita Suprinity (ZLS)	• Control<br>• Etching (9.5% HF, 20s ZLS /60s)<br>• Sandblasting (50 mm Al_2O_3 , 2 bar, 15s, 10 mm)<br>• Sandblasting + laser irradiation (Er:YAG 2,94 mm, 4W, 10 Hz, 400 mJ, 100 ms)<br>• Laser irradiation<br>• CoJet (15s, 2.8 bar, 10mm)	n = 10<br><br>2.5 mm	• Shear bond strength (SBS)	• Vita Suprinity - HF provided the highest SBS, being more effective than CoJet and sandblasting<br>• Vita YZ HT and inCoris TZI showed higher SBS when sandblasting and CoJet, compared to IPS e.max ZirCAD<br>• Laser irradiation did not show clinically acceptable SBS

Study	Study Groups	Surface treatments	Sample Size and Thicknes s	Parameters Assessed	Results
Maawadh et al., 2021	• Celtra Duo (CD)<br>• Vita Suprinity (ZLS)<br>• IPS e.max CAD	• Control: no etching<br>• Etching with 9% hydrofluoric acid (HF) for 10 s, 20 s, 30 s or 60 s<br>• The specimens were exposed to thermocycling for 5,000 cycles	n = 10<br><br>6 x 1.5 × 6 mm	• Shear bond strength (SBS)	• IPS e.max CAD etched for 20s showed higher SBS compared control, 10s, 30s and 60s groups<br>• CD with firing showed the highest SBS for 30s etching time<br>• CD fired had the lowest SBS in the control group<br>• ZLS showed the highest SBS when etched for 20-30s
Ataol et al., 2018	• IPS e.max CAD (Group A)<br>• IPS e.max ZirCAD (Group B)<br>• Vita Suprinity (Group C)	1 ST: HF + silane + bond<br>2 ST: alumina blasting + silane + Bond<br>3 ST: laser +silane + Bond<br>4 ST: HF + Primer<br>5 ST: alumina blasting + Primer<br>6 ST: laser + Primer<br>7 ST: silane + Bond (control)<br>8 ST: Primer (control) Half of the specimens were exposed to 5,000 thermocycling cycles	n = 14<br><br>12 × 12 × 3 mm	• Shear bond strength (SBS)	• Group A - HF etching showed higher SBS than the other groups; laser etching showed lower SBS than HF etching<br>• Group B - alumina blastingd had the highest SBS<br>• Group C - the highest SBS was achieved by HF etching

Study	Study Groups	Surface treatments	Sample Size and Thickness	Parameters Assessed	Results
Azevedo et al., 2021	• Vitablocs TriLuxe (FEL)<br>• Vita Suprinity PC (ZLS)	• Etching with 5% hydrofluoric acid (HF) for 20 s, 40 s or 60 s<br>• Etching with 10% hydrofluoric acid (HF) for 20 s, 40 s or 60 s<br>• The control group was sandblasted with 50 μm aluminum oxide, 20 s, 10 mm, 60 p	n = 20<br>10 x 5 x 1 mm^3	• Shear bond strength (SBS)	• ZLS - 10% HF (40 s) is the preferred surface treatment<br>• FEL - 5% or 10% HF (20 s) is the best surface treatment
Aladag et al., 2023	• Upcera ST (Y-TZP)<br>• Vita Suprinity (ZLS)<br>• Nice block (LD-LAS)	• Control<br>• Er:YAG (2940nm, 2W, 200mJ, 10Hz/s, 75μs)<br>• Nd:YAG (1064nm, 2W, 100mJ, 20Hz/s, 150μs)<br>• Etching with hydrofluoric acid (5% HFA, 20s)<br>• Er:YAG + HFA<br>• Nd:YAG + HFA<br>• Sandblasting (50 mm Al_2O_3 , 2 bar, 15s, 10 mm)	n = 7<br>2.5 mm	• Shear bond strength (SBS)	• Y-TZP - Er:YAG and Nd:YAG showed the highest SBS, as HFA had the lowest SBS<br>• ZLS - HFA, HFA + Er:YAG, and HFA + Nd:YAG showed significantly higher SBS compared to the control group<br>• ZLS - HFA + Er:YAG had the highest SBS; as the Er:YAG showed the lowest SBS.<br>• LD-LAS - HFA, HFA + Er:YAG, and HFA + Nd:YAG SBS

Study	Study Groups	Surface treatments	Sample Size and Thickness	Parameters Assessed	Results
					were higher than control group
Weyhrauch et al., 2016	• Vita Mark II (FSC)<br>• IPS Empress CAD (LrGC)<br>• IPS e.max CAD (LiDS)<br>• Vita Suprinity (PSZirLS)<br>• Vita Enamic (PolyFSP)<br>• Lava Ultimate (ResNC)<br>• Celtra Duo (FcZirLS)	• The implant abutments exterior surfaces were airborne-particle abraded - 50 µm, 10 mm, 1 bar, 60 s and silanized<br>• The crowns were etched with hydrofluoric acid or sandblasted and silanized<br>• The adhesion surface of the abutment and inner crown surfaces were also conditioned with silane	n = 10<br><br>14 mm block size	• Fracture strength	• LiDS, PSZirLS, PolyFSP and ResNC showed significantly higher fracture strength compared with FSP, FcZirLS, and LrGC<br>• PSZirLS showed the most increased fracture strength

**Study**	**Study Groups**	**Surface treatments**	**Sample Size and Thicknes s**	**Parameters Assessed**	**Results**
		• The specimens were exposed to 5,000 thermal cycles			
Sevmez et al., 2021	• Celtra Duo (CD)<br>• Vita Suprinity (VS)<br>• Vita Enamic (VE)<br>• Nacera Hybrid (NH)	• K: Control<br>H: 5% HF acid, 20s<br>• HS: 5% HF acid, 20s + silane 60s<br>• A: Air-abrasion, 50-micron Al2O3, 2.5 bar<br>AS: Air-abrasion, 50-micron Al2O3, 2.5 b pressure + silane 60s<br>C: Air-abrasion 30-micron silica-coated alumina 2.5 bar<br>• CS: Air-abrasion with 30-micron silica-coated alumina 2.5 bar + silane 60 s	n = 10<br><br>10 x 10 x 1 mm	• Shear bond strength (SBS)	• CD, VS and NH showed the highest SBS for H and HS groups<br>• VE had the highest SBS for CS group<br>• C and CS groups had the highest SBS for VE, and A and AS for VE and VS<br>• CD showed the lowest SBS for K group

Study	Study Groups	Surface treatments	Sample Size and Thickness	Parameters Assessed	Results
		• S: Silane 60 s			
Secilmis et al., 2016	• IPS e.max CAD (LDG)<br>• Vita Suprinity (ZLDC)<br>• Lava Ultimate (RNC)<br>• Vita Enamic (HC)	• RNC - 50µm aluminum oxide, 2 bar, 10 s, 10 mm)<br>• LDG and ZLDC - 9% HF, 20 s<br>• HC - HF, 60 s<br>• Multilink N (M)<br>• Panavia F 2.0 (P)	n = 12<br><br>2.0 mm blocs	• Shear bond strength (SBS)	• RNC-P showed the highest SBS<br>• LDG-M and ZLDC-M showed the highest bond strength<br>• ZLDC-P, HC-P, and LDG-P showed no significant differences<br>• RNC-M and HC-M showed no significant differences

Study	Study Groups	Surface treatments	Sample Size and Thickness	Parameters Assessed	Results
Traini et al., 2016	• Vita Suprinity (ZLS)	• The crystallization was done at 840°C for 8 min in a ceramic furnace<br>• Etching with 4,9% hydrofluoric acid (HF) for 20 s and 40 s<br>• Etching with 9,5% hydrofluoric acid (HF) for 20 s and 40 s	n = 8<br><br>3.5×3.0×1.0 mm	• Vickers hardness (Hv)<br>• Fracture toughness (Ft)	• Ft and HV values significantly increase after crystallization treatment of ZLS<br>• Etching with 4,9%HF for 20 s provided the best surface treatment
Donmez et al., 2020	• Vita Enamic<br>• IPS Empress CAD<br>• IPS e.max CAD<br>• Vita Suprinity	• Control<br>• Etching with MEP for 60 s<br>• Etching with MEP for 120 s<br>• Etching with 5% HF for 60s and 120s<br>• Etching with 9.5% HF for 60s and 120s<br>• The specimens were exposed to 5,000 thermocycling cycles	n = 10	• Shear bond strength (SBS)	• Hybrid ceramic showed a lower SBS than lithium disilicate glass-ceramic<br>• 9.5% HF showed higher BSB, regardless the exposure time<br>• Zirconia-reinforced lithium silicate showed no difference between the specimens treated with MEP60, HF-5%60s and HF-5%120s

**Study**	**Study Groups**	**Surface treatments**	**Sample Size and Thickness**	**Parameters Assessed**	**Results**
Al-Thagafi et al., 2016	• IPS e.max CAD<br>• Vita Suprinity	• Group C: control<br>• Group HF: 5%HF, 60s<br>• Group HF+H: 5%HF,60s and Heliobond<br>• Group CO: CoJet (tribochemical silica coating) 10 mm, 2.8 bar, 15s + Silane<br>• the specimens were thermocycled for 5,000 cycles	4 x 6 x 3 mm	• Microtensile Bond Strength (μTBS)	• IPS e.max CAD - group CO showed the highest and HF the lowest μTBS.<br>• Vita Suprinity - group CO exhibit the highest μTBS, and the lowest in HF and HF-H groups
Pucci et al., 2022	• Vita Enamic (PICN)<br>• Celtra Duo (ZLS)	Group 1: 5% HF + silanisation (SI)<br>Group 2: air-abrasion (AB) with 50 μm Al2O3 + 5% HF + SI<br>Group 3: 5% HF + universal adhesive (UA)<br>Group 4: air-abrasion (AB) with 50 μm Al2O3 + 5% HF + SI + UA<br>* HF- etching time - 30 s (ZLS) or 60 s (PICN)	n = 10<br><br>10 × 5 × 1.5 mm	• Shear bond strength (SBS)	• PICN treated with HF+UA presented the lowest SBS reduction and no differences at one year evaluation<br>• PICN and ZLS showed no differences at 24h, regardless the surface treatment<br>• PICN treated with HF+UA and ZLS treated with AB+HF+SI+UA showed higher SBS at one year

Study	Study Groups	Surface treatments	Sample Size and Thickness	Parameters Assessed	Results
Okutan et al., 2022	• IPS Empress CAD (EC)<br>• Vita Suprinity (VS)<br>• Vita Enamic (VE)	• Single-step self-etching system Monobond Etch & Prime (MEP) for 60 s	n = 10<br><br>1.0, 2.0 and 3.0 mm	• Shear bond strength (SBS)	• VS and VE had significantly lower SBS values than EC<br>• 2 mm EC and VS had higher SBS values than their 3 mm groups<br>• 2 mm and 3 mm specimens had significantly lower SBS values than 1 mm-thick
Preis et al., 2015	• Celtra Duo (ZLS)<br>• IPS e.max CAD (LDS)	• Etching with 5% hydrofluoric acid (HF) for 20 s LDS and 30 s ZLS<br>• Glass-ionomer cements (GIC)<br>• Resin and resin-modified self-adhesive cements<br>• TCML: 3000×5◦C/3000×55◦ C; 1.2 × 106 cycles, 50 N	n = 8<br><br>Crown:<br>• 1.5 mm circular and occlusal<br>• 1.0 mm circular shoulder	• Fracture resistance<br>• Marginal adaptation	• No significant differences were found between the cementation methods<br>• Marginal quality adaptation was found before and after TCML for ZLS and LDS<br>• Adhesive bonded ZLS showed a high fracture resistance, but their marginal quality decreased by TCML

Study	Study Groups	Surface treatments	Sample Size and Thickness	Parameters Assessed	Results
Falachai et al., 2020	• Vita Suprinity (ZLS)	• Etching with 5% hydrofluoric acid for 20 s<br>• Dual-cure resin cement (Panavia F2.0)<br>• 50-μm cement space	n = 10<br><br>Overlay preparation:<br>O, OS, OG and OSG	• Marginal adaptation	• group O showed significantly higher marginal adaptation than the group OSG<br>• No significant difference was noted in marginal adaptation of groups O, OS, and OG
Amini et al., 2021	• Celtra Duo (ZLS)<br>• DD Bio ZX2 (Zr)	• ZLS - hydrofluoric cis and silane<br>• Zr - sandblasting with 50 μm Al2O3<br>• Panavia F2.0 cement<br>• The specimens were exposed to 5000 thermomechanical cycles<br>• Cement space - 0 for the margins and 50 μm for other areas	n = 14<br><br>-2 mm occlusal<br>-3-5 mm pulp chamber deep<br>-6 degree divergence of the walls<br>-90 degree butt margin	• Marginal and internal adaptation	• ZLS showed significantly higher internal adaptation compared to the Zr<br>• For ZLS and Zr, cementation process and thermomechanical aging increased the marginal gap

Study	Study Groups	Surface treatments	Sample Size and Thickness	Parameters Assessed	Results
Vasiliu et al., 2020	• Vita Suprinity (ZLSM)<br>• Vita Mark II (FM)<br>• Vita PM9 (FP)<br>• Centra Press (ZLSP)	• Self-etch self-adhesive resin cement (Maxcem Elite, Self Etch, Kerr)<br>• The specimens were exposed to 10,000 thermal cycles	n = 8<br><br>-1.5 -2 mm occlusal<br>-1 mm rounded chamfer<br>-6 degree divergence of the walls<br>-lingual cusp beveled	• Marginal and internal adaptation	• ZLSP and ZLSM showed significant differences in the axial and occlusal areas<br>• FM showed the best marginal and internal fit<br>• After cementation and aging, FM showed decreased marginal fit compared to ZLS ceramics<br>• As, differences in the axial and occlusal areas were found between ZLSMP and ZLSP
Taha et al., 2018	• IPS e.max CAD (EX)<br>• Vita Enamic (VE)<br>• Celtra Duo (CD)<br>• Cerasmart (CS)		n = 10<br>Endocrown:<br>- 90 butt margin design<br>- 2 mm occlusal<br>- 6 mm extending into the pulp chamber	• Fracture resistance<br>• Marginal adaptation	• CS showed the highest mean fracture load with statistically significant difference than VE and CD<br>• CS and EX showed statistically insignificant differences<br>• Cementation and thermomechanic

Study	Study Groups	Surface treatments	Sample Size and Thicknes s	Parameters Assessed	Results
			- 8 degree divergenc e of the walls.		al aging increased statistically significant the marginal gap, for all the materials
Alves et al., 2020	IPS e.max CAD (LD)<br>Vita Suprinity (ZLS)<br>Prettau Anterior (Trans YZ)	LD and ZLS - 5% Hydrofluoric acid applied for 20s and silane<br>Trans YZ - Airborne-particle abrasion with 45 μm aluminum oxide particles, 15 s and silane	n = 15<br><br>Crown design: flat occlusal surface 1.5 mm for all regions	Fatigue	Trans YZ material showed the highest fatigue performance, and survival rate followed by ZLS and LD<br>ZLS showed superior fatigue resistance to LD
Sahebi et al., 2022	Celtra Duo (ZLS)<br>DD BIO ZX2 (ZR)	Zr - air-abrasion with 50 μm alumina particles<br>ZLS - 9% hydrofluoric acid (HF), 30 s + silane<br>Specimens were subjected to 5000 thermomechanical cycles	n = 14<br><br>-2 mm occlusal<br>-4 mm pulp chamber deep<br>-6 degree divergenc e of the walls	Fracture load<br>Retention test	ZR endocrowns showed higher fracture loads than ZLS endocrowns<br>ZLS had higher retention compared to ZR endocrowns

Study	Study Groups	Surface treatments	Sample Size and Thicknes s	Parameters Assessed	Results
			-90 degree butt margin		
